# Interaction of Sleep Duration and Sleep Quality on Hypertension Prevalence in Adult Chinese Males

**DOI:** 10.2188/jea.JE20140139

**Published:** 2015-06-05

**Authors:** Kai Lu, Jia Chen, Shouling Wu, Ji Chen, Dayi Hu

**Affiliations:** 1 Department of Cardiology, The First Affiliated Hospital of Chongqing Medical University, Chongqing, China; 2 Kailuan General Hospital, Hebei United University, Tangshan, China

**Keywords:** sleep duration, sleep quality, hypertension

## Abstract

**Background:**

Previous studies demonstrated conflicting results about the association of sleep duration and hypertension. Given the potential relationship between sleep quality and hypertension, this study aimed to investigate the interaction of self-reported sleep duration and sleep quality on hypertension prevalence in adult Chinese males.

**Methods:**

We undertook a cross-sectional analysis of 4144 male subjects. Sleep duration were measured by self-reported average sleep time during the past month. Sleep quality was evaluated using the standard Pittsburgh Sleep Quality Index. Hypertension was defined as blood pressure level ≥140/90 mm Hg or current antihypertensive treatment. The association between hypertension prevalence, sleep duration, and sleep quality was analyzed using logistic regression after adjusting for basic cardiovascular characteristics.

**Results:**

Sleep duration shorter than 8 hours was found to be associated with increased hypertension, with odds ratios and 95% confidence intervals (CIs) of 1.25 (95% CI, 1.03–1.52) for 7 hours, 1.41 (95% CI, 1.14–1.73) for 6 hours, and 2.38 (95% CI, 1.81–3.11) for <6 hours. Using very good sleep quality as the reference, good, poor, and very poor sleep quality were associated with hypertension, with odds ratios of 1.20 (95% CI, 1.01–1.42), 1.67 (95% CI, 1.32–2.11), and 2.32 (95% CI, 1.67–3.21), respectively. More importantly, further investigation of the association of different combinations of sleep duration and quality in relation to hypertension indicated an additive interaction.

**Conclusions:**

There is an additive interaction of poor sleep quality and short sleep duration on hypertension prevalence. More comprehensive measurement of sleep should be performed in future studies.

## INTRODUCTION

The association between sleep disorders and hypertension has aroused the attention of cardiologists for a long time. Many prospective studies have suggested a robust relationship between short sleep duration and hypertension risk.^1^^–^^5^ However, sleep consists of both qualitative and quantitative aspects, and two previous studies suggested that maybe it is not enough to evaluate sleep only by measuring sleep duration when investigating the potential relationship between sleep and hypertension.^6^^,^^7^ In recent years, the potential associations between sleep quality and several cardiovascular risk factors have been explored in several cross-sectional studies, and the results suggested that sleep quality was associated with prevalence of metabolic syndrome and obesity.^8^^–^^12^ Poor sleep quality was also found to have an adverse effect on fasting blood glucose control.^13^^,^^14^ Hypertension shares many common potential mechanisms with cardiometabolic disorders,^15^ but the specific association of sleep quality and hypertension prevalence is still inconclusive.^4^^,^^16^ In addition, considering the fact that people with short sleep duration often have a high prevalence of poor sleep quality,^17^ it is necessary to preclude the potential interactively confounding effects of both sleep duration and sleep quality and confirm the specific and separate roles of sleep quality and duration in hypertension prevalence.

In this study, we investigated the potential association of self-reported sleep duration and quality in relation to hypertension prevalence in adult Chinese males using the data from a cross-sectional survey. Further, the interaction of sleep duration and quality on hypertension prevalence was explored.

## MATERIALS AND METHODS

### Study design and population

This study was designed as a cross-sectional study and was conducted from September to December 2013 in Fangezhuang, Tangshan, Lvjiatuo, and Qianjiaying communities located in the northern China city of Tangshan, which is approximately 180 km southeast of the capital of China. Subjects aged 18 years or older in the 4 communities were invited to participate in this study. Critical exclusion criteria included those with a previous diagnosis of obstructive sleep apnea syndrome (OSAS) or restless legs syndrome (RLS), as well as those who reported snoring by themselves or roommates. The contents and purposes of this study were thoroughly explained to the participants prior to the study, and written consent was obtained. The study protocol was in accordance with the Declaration of Helsinki, and ethical approval was obtained from the Science and Technology Committee of Tangshan City.

Citizens in the Fangezhuang, Tangshan, Lvjiatuo, and Qianjiaying communities are mainly employees of the Kailuan Group, a large-scale comprehensive enterprise that mainly manages coal products and has a higher male to female ratio than the general Chinese population. Only 571 female citizens were enrolled in this study, a sample size too small for subsequent statistical analysis; we therefore only presented the relevant results from male participants in the current report.

### Anthropometric measurements

Doctors and nurses were trained in the standard protocol of measurement before the survey. Height and weight were measured to the nearest 0.1 cm and 0.1 kg, respectively, when the subjects stood upright and were barefoot in light clothes. Two separate measurements were performed for each subject, and the average was used for analysis. BMI was calculated as the ratio of weight (kg) to height (m) squared (kg/m^2^). Blood pressure was measured in a sitting position with calibrated standard mercury sphygmomanometer (Yuyue Medical Equipment & Supply Co., Ltd., Jiangsu, China), and an average of two readings was used in the present study. If the two readings differed by more than 5 mm Hg, a third reading was taken, and the average of three readings was used. Hypertension was defined according to the 7th edition report of the American Joint National Committee on Prevention, Detection, Evaluation, and Treatment of Hypertension^18^ as SBP ≥140 mm Hg and/or DBP ≥90 mm Hg on average of measurements or by current antihypertensive treatment according to hospital records.

### Blood test

Subjects were asked to fast overnight before venous blood sample collection. Plasma samples were prepared by centrifuging at 3000 rpm for 10 minutes within 4 hours of blood collection for determination of total cholesterol (TC) and fasting blood glucose (FBG) in the central laboratory of Kailuan Hospital on automatic biochemical analyzers (Hitachi 717; Hitachi, Tokyo, Japan).

### Questionnaire

A structured questionnaire was administered face to face to each subject and recorded on paper to obtain demographic and behavior-associated information, including age, gender, smoking status, drinking status, educational level, physical activity, sleep duration, and sleep quality. Smoking and drinking status were classified using self-reported information as “never”, “former”, or “current”. Subjects who consumed more than 175 grams of alcohol per week in the past half year were defined as current drinkers. Physical activity was evaluated from responses to questions about the type and frequency of physical activity during leisure time. Individuals were classified as “active” and “inactive” according to whether or not at least 30 minutes aerobic exercise for at least 5 days per week was attained. Educational level was assessed by responses to questions regarding the final degree attained, and senior high school or higher was defined as well-educated. Sleep duration was evaluated from the responses to questions about average sleep duration in the past month, and participants were reminded that time spent awake in bed was not included. Sleep duration was categorized into “<6 hours”, “6 hours”, “7 hours”, “8 hours”, and “>8 hours”. Sleep quality was evaluated using the standard Pittsburgh Sleep Quality Index (PSQI), which is a widely used measure of sleep quality,^19^ and sleep quality was classified as “very good” (score <3 on PSQI), “good” (score 3 to <6 on PSQI), “poor” (score of 6 to <9 on PSQI), and “very poor” (score ≥9 on PSQI). The English version of the PSQI and the scoring system are provided in eAppendix 1.

In addition, considering the frequent comorbidity of sleep disorders with anxiety and depression, anxiety and depression status of participants was evaluated using the General Anxiety Disorder-7 (GAD-7) and Patient Health Questionnaire-9 (PHQ-9 scales, respectively. GAD-7 is a seven-question inventory for self-assessment and is one of the most common instruments for measuring severity of anxiety.^20^ PHQ-9 is a widely used nine-question inventory for self-assessment of depression.^21^

### Statistical analysis

Continuous variables were presented as mean (standard error [SE]) and categorical variables as frequency (proportion). Continuous variables were compared using one-way ANOVA followed by Dunnetts’s post-hoc test, and categorical variables were compared using the χ^2^ test. The association between sleep duration, sleep quality, and hypertension prevalence was investigated by logistic regression analysis, and we adjusted for plausible confounders, including age, BMI, smoking status, drinking status, physical activity, educational level, and anxiety and depression scores. Further, to investigate the interaction of sleep duration and sleep quality on hypertension prevalence, participants were divided into groups of different combinations of sleep duration and sleep quality. Odds ratios (ORs) and 95% confidence intervals (CIs) of each group were calculated using multiple logistic regression analysis, with the group of 8 hours’ sleep duration and very good sleep quality as the reference. For all comparisons, the level of statistical significance was set at *P* < 0.05 (two-sided). SPSS 19.0 (IBM, New York, USA) was used for all statistical analyses.

## RESULTS

### Basic characteristics

A total of 6120 citizens (98.9%) responded to our invitation, and 1926 (31.4%) of them were excluded due to report of OSAS (*n* = 351, 5.7%), RLS (*n* = 43, 0.7%), or snoring (*n* = 1532, 25.0%). A total of 4144 male subjects were finally enrolled in this study, and the basic characteristics of them are presented in Table 1 and Table 2 according to sleep duration and sleep quality. The numbers of subjects with sleep duration of <6 hours, 6 hours, 7 hours, 8 hours, and >8 hours were 356 (8.6%), 1021 (24.6%), 1446 (34.9%), 1082 (26.1%), and 239 (5.8%), respectively. The numbers of subjects with sleep quality of very good, good, poor, and very poor were 2369 (57.2%), 1146 (27.7%), 468 (11.3%), and 191 (4.6%), respectively. The average anxiety and depression scores and the percentage of current smokers, current drinkers, active exercisers, and well-educated subjects were significantly different in groups with different sleep durations or qualities.

**Table 1.** Basic characteristics of participants according to sleep duration in adult Chinese males

	Sleep duration (hours)						
	<6 (*n* = 356)	6 (*n* = 1021)	7 (*n* = 1446)	8 (*n* = 1082)	>8 (*n* = 239)	Total (*n* = 4144)	*P*
Age (years)	47.91 (0.37)	46.84 (0.23)	46.92 (0.18)	46.98 (0.41)	47.47 (0.42)	47.04 (0.14)	0.33
BMI (kg/m^2^)	25.45 (0.19)	25.17 (0.11)	25.44 (0.10)	25.19 (0.10)	25.05 (0.19)	25.28 (0.06)	0.16
SBP (mm Hg)	130.61 (0.71)	129.79 (0.47)	129.96 (0.38)	129.62 (0.44)	131.84 (0.81)	130.02 (0.22)	0.18
DBP (mm Hg)	85.87 (0.47)	85.9 (0.31)	85.80 (0.25)	85.71 (0.30)	86.86 (0.55)	85.88 (0.15)	0.50
Total cholesterol (mmol/L)	4.85 (0.05)	4.88 (0.03)	4.85 (0.02)	4.92 (0.03)	4.85 (0.05)	4.88 (0.01)	0.48
Fasting blood glucose (mmol/L)	5.57 (0.08)	5.41 (0.05)	5.43 (0.04)	5.50 (0.05)	5.29 (0.07)	5.45 (0.02)	0.08
Score of anxiety	4.40 (0.28)	3.35 (0.15)	1.63 (0.08)	1.51 (0.09)	1.93 (0.30)	2.27 (0.06)	0.00
Score of depression	5.06 (0.32)	3.29 (0.16)	2.02 (0.10)	1.63 (0.10)	2.25 (0.32)	2.48 (0.07)	0.00
Current smoker (%)	215 (60.5)	611 (59.8)	781 (54.0)	576 (53.2)	133 (55.8)	2316 (55.9)	0.00
Current drinker (%)	115 (32.2)	289 (28.3)	320 (22.1)	285 (26.2)	62 (25.9)	1071 (25.8)	0.00
Active exercise habit (%)	109 (30.6)	311 (30.5)	508 (35.1)	392 (36.2)	59 (24.6)	1379 (33.3)	0.00
Well educated (%)	140 (39.4)	415 (40.6)	450 (31.1)	413 (38.2)	92 (38.7)	1510 (36.4)	0.00

BMI, body mass index; SBP, systolic blood pressure; DBP, diastolic blood pressure.

**Table 2.** Basic characteristics of participants according to sleep quality in adult Chinese males

	Sleep quality					
	Very good (*n* = 2369)	Good (*n* = 1146)	Poor (*n* = 468)	Very poor (*n* = 191)	Total (*n* = 4144)	*P*
Age (years)	47.20 (0.14)	46.38 (0.40)	47.48 (0.36)	47.34 (0.52)	47.01 (0.14)	0.06
BMI (kg/m^2^)	25.26 (0.08)	25.33 (0.11)	25.41 (0.18)	25.01 (0.23)	25.29 (0.06)	0.58
SBP (mm Hg)	130.53 (0.30)	129.16 (0.42)	130.16 (0.69)	128.32 (1.04)	130.01 (0.23)	0.02
DBP (mm Hg)	86.24 (0.20)	85.32 (0.28)	85.69 (0.48)	85.38 (0.7)	85.89 (0.15)	0.05
Total cholesterol (mmol/L)	4.90 (0.02)	4.88 (0.03)	4.77 (0.04)	4.89 (0.06)	4.88 (0.01)	0.06
Fasting blood glucose (mmol/L)	5.47 (0.03)	5.44 (0.05)	5.38 (0.06)	5.42 (0.10)	5.45 (0.02)	0.68
Score of anxiety	1.15 (0.06)	3.06 (0.12)	4.54 (0.26)	6.4 (0.43)	2.27 (0.06)	0.00
Score of depression	1.21 (0.06)	3.54 (0.13)	4.60 (0.26)	7.9 (0.53)	2.48 (0.07)	0.00
Current smoker (%)	1279 (54.0)	662 (57.8)	256 (54.7)	119 (62.2)	2316 (55.9)	0.04
Current drinker (%)	572 (24.1)	289 (25.2)	132 (28.3)	78 (40.8)	1071 (25.8)	0.00
Active exercise habit (%)	785 (33.1)	416 (36.3)	126 (26.9)	52 (27.2)	1379 (33.3)	0.00
Well educated (%)	811 (34.2)	452 (39.4)	162 (34.7)	85 (44.4)	1510 (36.4)	0.00

BMI, body mass index; SBP, systolic blood pressure; DBP, diastolic blood pressure.

### Prevalence of hypertension

The prevalence of hypertension in subjects with different combinations of sleep duration and sleep quality are presented in Figure. Generally, a U-shaped relationship could be observed between sleep duration and hypertension prevalence. With the exception of those with very good sleep quality, subjects with sleep duration of 8 hours had the lowest prevalence of hypertension, and participants with both less and more sleep time had increased hypertension prevalence. However, the trend was not statistically significant. Figure also shows that hypertension prevalence increased with worsening sleep quality in those with different sleep duration, but this trend was also not statistically significant.

**Figure.**
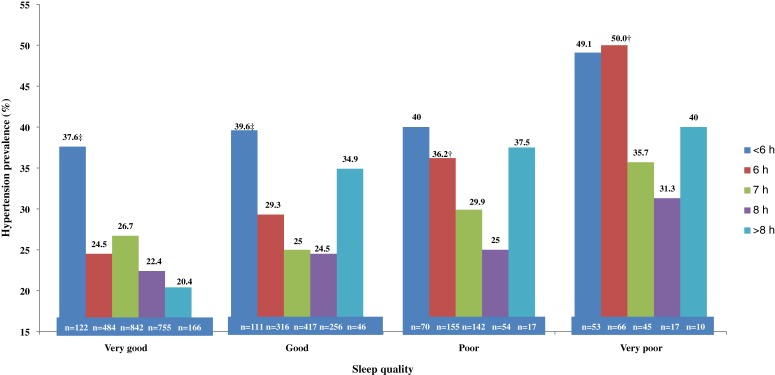
Hypertension prevalence in participants with different combinations of sleep duration and sleep quality

### Association of sleep duration or sleep quality in related to hypertension prevalence

The association between sleep duration or quality and hypertension prevalence was analyzed using logistic regression, and the results are presented in Table 3. With sleep duration of 8 hours as the reference, less sleep time was found to be associated with the prevalence of hypertension among Chinese males after adjustment of confounders, with an odds ratio of 1.25 (95% CI, 1.03–1.52) for 7 hours, 1.41 (95% CI, 1.14–1.73) for 6 hours, and 2.38 (95% CI, 1.81–3.11) for less than 6 hours. In contrast, no significant relationship was found between sleep duration of more than 8 hours and hypertension prevalence. In comparison to those with very good sleep quality, the ORs for subjects with good, poor, and very poor sleep quality were 1.20 (95% CI, 1.01–1.42), 1.67 (95% CI, 1.32–2.11) and 2.32 (95% CI, 1.67–3.21), respectively.

**Table 3.** Odds ratios and 95% confidence intervals of sleep duration and sleep quality for hypertension prevalence in adult Chinese males

		*n*	Unadjusted OR (95% CI)	*P*	Adjusted OR (95% CI)^b^	*P*
Sleep duration (hours)	<6	356	2.40 (1.86–3.11)	0.00	2.41 (1.85–3.18)	0.00
	6	1021	1.37 (1.13–1.67)	0.00	1.39 (1.14–1.69)	0.00
	7	1446	1.24 (1.03–1.49)	0.02	1.22 (1.02–1.47)	0.00
	8	1082	Reference	—	Reference	—
	>8	239	0.88 (0.64–1.20)	0.42	0.84 (0.61–1.17)	0.38

		*n*	Unadjusted OR (95% CI)	*P*	Adjusted OR (95% CI)^b^	*P*

Sleep quality^a^	Very good	2369	Reference	—	Reference	—
	Good	1146	1.16 (0.99–1.36)	0.08	1.15 (1.08–1.23)	0.04
	Poor	438	1.65 (1.33–2.06)	0.00	1.66 (1.32–2.09)	0.00
	Very poor	191	2.16 (1.59–2.93)	0.00	2.30 (1.68–3.17)	0.00

CI, confidence interval; OR, odds ratio.

^a^Sleep quality was evaluated by standard Pittsburgh Sleep Quality Index and is categorized based on total score.

^b^Adjusted for age, body mass index, status of smoking and drinking, exercise habit, educational level, score of anxiety and depression.

### Association of different combinations of sleep duration and sleep quality in relation to hypertension prevalence

The unadjusted and adjusted ORs for hypertension prevalence according to different combinations of sleep duration and sleep quality are presented in Table 4. In comparison to the those with 8 hours sleep duration and very good sleep quality (the reference group), subjects in the following groups had increased hypertension prevalence after adjusting for the basic cardiometabolic characteristics: <6 hours sleep duration combined with any sleep quality (OR 2.27 [95% CI, 1.46–3.53] for very good; OR 2.54 [95% CI, 1.60–4.04] for good; OR 3.42 [95% CI, 2.21–5.30] for poor or very poor), 6 hours sleep duration combined with good, poor, or very poor sleep (OR 1.53 [95% CI, 1.10–2.13] for good; OR 2.40 [95% CI, 1.65–3.49] for poor or very poor), and 7 hours sleep duration combined with poor or very poor sleep (OR 1.68 [95% CI, 1.13–2.51]).

**Table 4.** Odds ratios and 95% confidence intervals for hypertension prevalence according to different combinations of sleep duration and quality in adult Chinese males

		Sleep duration (hours)							

		<6		6		7		8	
Unadjusted		*n*	OR (95% CI)	*n*	OR (95% CI)	*n*	OR (95% CI)	*n*	OR (95% CI)

Sleep quality^a^	Very good	**122**	**2.09 (1.38–3.16)**	484	1.27 (0.99–1.60)	842	1.13 (0.86–1.48)	755	Reference
	Good	**111**	**2.28 (1.49–3.49)**	**316**	**1.44 (1.07–1.94)**	417	1.16 (0.87–1.54)	256	1.13 (0.80–1.58)
	Poor or very poor	**123**	**3.25 (2.18–4.83)**	**221**	**2.35 (1.70–3.25)**	**187**	**1.58 (1.10–2.27)**	71	1.25 (0.71–2.20)

Adjusted^b^		*n*	OR (95% CI)	*n*	OR (95% CI)	*n*	OR (95% CI)	*n*	OR (95% CI)

Sleep quality^a^	Very good	**122**	**2.27 (1.46–3.53)**	484	1.27 (0.98–1.63)	842	1.18 (0.88–1.58)	755	Reference
	Good	**111**	**2.54 (1.60–4.04)**	**316**	**1.53 (1.10–2.13)**	417	1.21 (0.89–1.64)	256	1.14 (0.79–1.66)
	Poor or very poor	**123**	**3.42 (2.21–5.30)**	**221**	**2.40 (1.65–3.49)**	**187**	**1.68 (1.13–2.51)**	71	1.39 (0.76–2.54)

CI, confidence interval; OR, odds ratio.

^a^Sleep quality was evaluated by standard Pittsburgh Sleep Quality Index and is categorized based on total score.

^b^Adjusted for age, body mass index, status of smoking and drinking, exercise habit, educational level, score of anxiety and depression.

## DISCUSSION

In this cross-sectional study, we investigated the separate and combined association of sleep duration and sleep quality with the prevalence of hypertension in adult Chinese males. Shorter sleep duration than normal was found to be associated with hypertension prevalence, but not longer sleep duration. In addition, poor sleep quality was also associated with hypertension prevalence. The present study investigated the association of different combinations of sleep duration and sleep quality in relation to hypertension prevalence, and the results indicated an additive interaction between sleep quality and duration and hypertension prevalence.

It has been argued that the relationship between sleep and hypertension maybe gender-specific. Two prospective studies conducted in English and Korean populations^22^^,^^23^ and several cross-sectional studies^22^^,^^24^^–^^26^ have demonstrated that short sleep duration was associated with hypertension incidence only in women. The underlying mechanisms that explain sex-specific correlation of short sleep duration and hypertension are still unknown, although sex differences in hormone secretion, stress responses, inflammatory reaction, and changes in sympathetic nerve activity have been suggested.^27^^–^^30^ Though the current literature supports a more robust correlation between short sleep duration and hypertension in women than men, one cross-sectional study by Fang et al indicated the possibility of a complex association between sleep duration and hypertension.^31^ The present cross-sectional study adds new evidence concerning the association between sleep duration and hypertension in men. However, we still hold a cautious attitude towards the gender-specific association mentioned above because we think only measuring sleep duration in the previous studies is not sufficient to assess the global sleep status, and results are somewhat unreliable.

Two previous studies have suggested a U-shaped relationship between sleep duration and hypertension in adults and adolescents, which meant not only short but also long sleep duration was related to hypertension prevalence and incidence.^32^^,^^33^ In the current study, we also observed a U-shaped trend between sleep duration and hypertension prevalence, but it failed to reach statistical significance. One of the findings of the current study was that sleep duration and quality were additively related to hypertension. Neglecting the potential role of sleep quality may explain the conflicting results, but we must note that the small sample size of subjects with longer sleep duration may make our results less persuasive.

Sleep has both qualitative and quantitative aspects. Two studies have demonstrated that short sleep duration only failed to increase hypertension risk, but a combination of sleep duration and other sleep disorders did increase risk, which indicated that evaluation of sleep only by measuring sleep duration was not sufficient.^6^^,^^7^ This viewpoint was supported by the current study. Sleep quality was measured by the PSQI in this study, and the results show that sleep quality was also significantly correlated with hypertension risk. Bruno et al investigated the relationship between sleep quality assessed by the PSQI and resistant hypertension, and their results were consistent with ours.^34^ Another study also indicated a relationship between sleep quality and blood pressure level, although sleep quality was assessed by overnight polysomnography in that study.^35^

We must note that people with sleep insufficiency often have poor sleep quality. Therefore, to preclude the interactively confounding effect of sleep duration and quality in the current study, the separate and combined associations of sleep duration and sleep quality in relation to hypertension were explored. The results demonstrated that the association of sleep duration and sleep quality with hypertension was an additive relationship. For example, no category of sleep quality was found to be related to hypertension prevalence among those with normal sleep duration. However, among those with sleep duration less than 6 hours, each category of sleep quality was related to hypertension prevalence. In contrast, for those with 7 hours sleep, only poor or very poor sleep quality was found to be associated with hypertension. The current study suggests an approximately equal contribution of sleep quality and sleep duration to hypertension prevalence. Failing to pay attention to the effect of sleep quality may explain the conflicting results among previous studies about the relationship between sleep duration and hypertension prevalence or development.^1^^,^^22^^,^^36^^–^^38^

OSAS and RLS are possible confounding factors in the relationship between sleep status and hypertension prevalence,^39^^,^^40^ and it was necessary to preclude the potential effects of these conditions in the current study. The diagnosis of OSAS was based on the results of polysomnography, but the test was very difficult to perform for each subject due to the limited funds and time. Therefore, we adopted a comparatively easy way to preclude OSAS according to whether or not the subject snored during sleep, which was reported by the subjects themselves or by their roommates. It has been reported that snoring has a high sensitivity (87%) for detecting OSAS.^41^ In addition, RLS was excluded on the basis of self-reported (roommate-reported) symptoms, considering that the clinical diagnosis of RLS was mainly based on self-reported symptoms.^42^

In the current study, we paid attention to several potential confounders for the association between sleep and hypertension, such as educational level, anxiety, and depression, which were rarely controlled for in previous studies. Socio-economic status is a well-known risk factor for hypertension.^43^ Recently, mental status, such as anxiety and depression, has also been suggested to be related to onset and control of hypertension.^44^^,^^45^ Considering the frequent comorbidity of anxiety and depression in sleep disorders and the effect of educational level on sleep,^5^ it is necessary to control for the confounding effect of these factors, as we have done in the present study.

Possible mechanisms accounting for the association between sleep and hypertension have been reported in a couple of previous studies, although they are far from being fully elucidated. Most of those studies support that sympathetic overactivity due to sleep deprivation was associated with elevated blood pressure level.^46^^–^^48^ Additionally, as a contributor to psychological stress, sleep insufficiency could also induce sodium retention, proinflammatory responses, and endothelial dysfunction through the activation of the neuroendocrine system.^28^^,^^48^^,^^49^

Due to the cross-sectional design of the present study, we cannot assess the causal relationship between the two sleep aspects and hypertension prevalence. However, the hypothesis that sleep deprivation is involved in the development and sustainment of hypertension has been better established than the opposite cause-effect link. Although it is possible that living with hypertension, which acts as a mental stressor, may disturb sleep homeostasis directly or through drug treatments (such as diuretics) that are often prescribed for hypertensive patients, rare relevant evidence is available in the current literature.^50^^,^^51^ In addition, Bruno et al analyzed the association of sleep quality and the most frequently prescribed antihypertensive agents and found no relationship between them.^34^

Our study has several limitations. First of all, it has been reported that logistic analysis used in cross-sectional study may overestimate the prevalence ratio, although it is still the most frequently used method in studies with a similar design to ours.^52^ Second, there are no standard cut-off values to judge good or poor sleep quality, and the cut-off values used in this study were based on previous reports and our experience. Third, eating habit is one important meditating factor in the relationship between sleep disorders and increased prevalence of hypertension, but we did not investigate this because we did not have suitable questionnaires about eating habits.

Despite the above limitations, this cross-sectional study demonstrates for the first time that both short sleep duration and poor sleep quality are associated with hypertension prevalence in adult Chinese males. More importantly, the current study suggests that the association of sleep duration and quality with hypertension is an additive relationship.

## ONLINE ONLY MATERIAL

eAppendix 1. The Pittsburgh Sleep Quality Index (PSQI).
