# Supplementary material for: Interaction of Sleep Duration and Sleep Quality on Hypertension Prevalence in Adult Chinese Males
Source: J Epidemiol. 2015 Jun 5;25(6):415–22. doi: 10.2188/jea.JE20140139 (PMC4444495; doi:10.2188/jea.JE20140139)
Supplement: eAppendix 1. [file je-25-415-s001.pdf]

The Pittsburgh Sleep Quality Index (PSQI)©

Instructions: The following questions relate to your usual sleep habits during the past month only. Your answers should indicate the most accurate reply for the majority of days and nights in the past month. Please answer all questions. During the past month…

1. When have you usually gone to bed? ______________
2. How long (in minutes) has it taken you to fall asleep each night? ______________
3. When have you usually gotten up in the morning? ______________
4. How many hours of actual sleep do you get at night? (This may be different than the number of hours you spend in bed) ______________

5. During the past month, how often have you had trouble sleeping because you	Not during the past month (0)	Less than once a week (1)	Once or twice a week (2)	Three or more times a week (3)
a. Cannot get to sleep within 30 minutes				
b. Wake up in the middle of the night or early morning				
c. Have to get up to use the bathroom				
d. Cannot breathe comfortably				
e. Cough or snore loudly				
f. Feel too cold				
g. Feel too hot				
h. Have bad dreams				
i. Have pain				
j. Other reason(s), please describe, including how often you have had trouble sleeping because of this reason(s):				
6. During the past month, how often have you taken medicine (prescribed or "over the counter") to help you sleep?				
7. During the past month, how often have you had trouble staying awake while driving, eating meals, or engaging in social activity?				
8. During the past month, how much of a problem has it been for you to keep up enthusiasm to get things done?				
	Very good (0)	Fairly good (1)	Fairly bad (2)	Very bad (3)
9. During the past month, how would you rate your sleep quality overall?				

Component 1	#9 Score..	C1_______
Component 2	#2 Score (″15min=0; 16-30 min=1; 31-60 min=2, >60 min=3) + #5a Score<br>(if sum is equal 0=0; 1-2=1; 3-4=2; 5-6=3)	C2_______
Component 3	#4 Score (>7=0; 6-7=1; 5-6=2; <5=3)	C3_______
Component 4	(total # of hours asleep)/(total # of hours in bed) x 100<br>>85%=0, 75%-84%=1, 65%-74%=2, <65%=3	C4_______
Component 5	Sum of Scores #5b to #5j (0=0; 1-9=1; 10-18=2; 19-27=3)................................	C5_______
Component 6	#6 Score	C6_______
Component 7	#7 Score + #8 Score (0=0; 1-2=1; 3-4=2; 5-6=3)..	C7_______

Add the seven component scores together _______ Global PSQI Score _______

Reproduced with permission.
